# Novel Brain Protection Method for Zone 0 Endovascular Aortic Repair with Selective Cerebral Perfusion

**DOI:** 10.3400/avd.oa.21-00025

**Published:** 2021-06-25

**Authors:** Ryuta Seguchi, Ryuta Kiuchi, Takafumi Horikawa, Tatsuya Tarui, Junichiro Sanada, Hiroshi Ohtake, Go Watanabe

**Affiliations:** 1 Department of Cardiovascular Surgery, NewHeart Watanabe Institute, Tokyo, Japan; 2 Department of Vascular Surgery, Ageo Central General Hospital, Ageo, Saitama, Japan

**Keywords:** brain protection, endovascular aortic repair, zone 0, extracorporeal membrane oxygenation

## Abstract

**Objective:** Zone 0 thoracic endovascular aortic repair (TEVAR) is associated with a high incidence of cerebral infarction mostly due to the embolic shower of a plaque from the aortic arch when the stent graft brushes against the aortic wall. Thus, it is important to develop a method for protecting the brain from such embolism. We report the outcomes of Zone 0 TEVAR with a novel brain protection method using selective cerebral perfusion under extracorporeal membrane oxygenation (ECMO).

**Materials and Methods:** Two T-shaped grafts with ringed expanded polytetrafluoroethylene (ePTFE) were created using an 8-mm-ringed ePTFE anastomosed end-to-side with a 7-mm-ringed ePTFE. Carotid–carotid bypass and axillo-axillary bypass were established using these grafts. ECMO was connected to the grafts and the femoral vein. Bilateral carotid and axillary arteries were blocked, and cerebral perfusion was selectively maintained using ECMO. Total endovascular Zone 0 TEVAR was performed. The patency of brachiocephalic artery was maintained using the chimney or in situ fenestration technique.

**Results:** Since August 2016, seven patients with aortic arch aneurysms underwent the procedure. The mortality rate was 0%. No neurological complications developed.

**Conclusion:** This brain protection method using selective cerebral perfusion under ECMO is a safe method for Zone 0 TEVAR.

## Introduction

A complication that cannot be ignored in the surgeries of aortic arch aneurysms is cerebral infarction. The rates of neurological complications and mortality following open total arch replacement are approximately 5%–11% and 6%–17%, respectively.^1)^ Thoracic endovascular aortic repair (TEVAR) has rapidly emerged. With its potential to avoid median sternotomy, cardiac arrest, and aortic cross-clamping, TEVAR has substantially improved patient outcomes in terms of reduced mortality and rate of paraplegia and shorter stays in the hospital and intensive care unit (ICU).^2)^ However, the risk varies depending on the location of the proximal landing zone. Ishimaru described the ascending aorta as Zone 0.^3)^ The incidence of stroke in the Zone 0 landing is approximately 4%–27% and is associated with a high mortality rate.^2,4,5)^ It is mostly due to the embolic shower of plaque from the aortic arch when the stent graft or the stiff guidewire brushes against the aortic wall.^4,6)^ To protect the brain from ischemia, both the anterior circulation from the carotid arteries and the posterior circulation from the vertebral arteries need to be protected from embolic shower. We developed a method for protecting the brain from embolism by completely separating the cerebral and systemic circulations along with the support of extracorporeal membrane oxygenation (ECMO). We herein report the outcomes of percutaneous Zone 0 TEVAR with this novel brain protection method using selective cerebral perfusion under cardiopulmonary support.

## Patients and Methods

Between August 2016 and December 2019, seven patients underwent Zone 0 TEVAR without opening of the chest using the brain protection method. Selective cerebral perfusion under cardiopulmonary support was adopted. **Table 1** presents the preoperative patient characteristics. This study was approved by the ethics committee of NewHeart Watanabe Institute, Japan (Approval Number 20210002).

**Table 1:** Table 1 Preoperative patient characteristics (n=7)

Age (years)	80.3±6.0
Male (%)	6 (86)
Comorbidities	
Hypertension (%)	7 (100)
Hyperlipidemia	4 (57)
Diabetes mellitus	1 (14)
History of CVA	3 (43)
COPD (%)	5 (71)
Hemodialysis	0 (0)
LVEF (%)	65.0±5.5
Creatinine (mg/dl)	0.93±0.15
CT grading of aortic atheroma	
Grade I (%)	0 (0)
Grade II (%)	1 (14)
Grade III (%)	3 (43)
Grade IV (%)	3 (43)

Data expressed as n (%) or mean±standard deviation. CVA: cerebrovascular accident; COPD: chronic obstructive pulmonary disease; LVEF: left ventricular ejection fraction; CT: computed tomography

### Surgical methods

**Figure 1:**
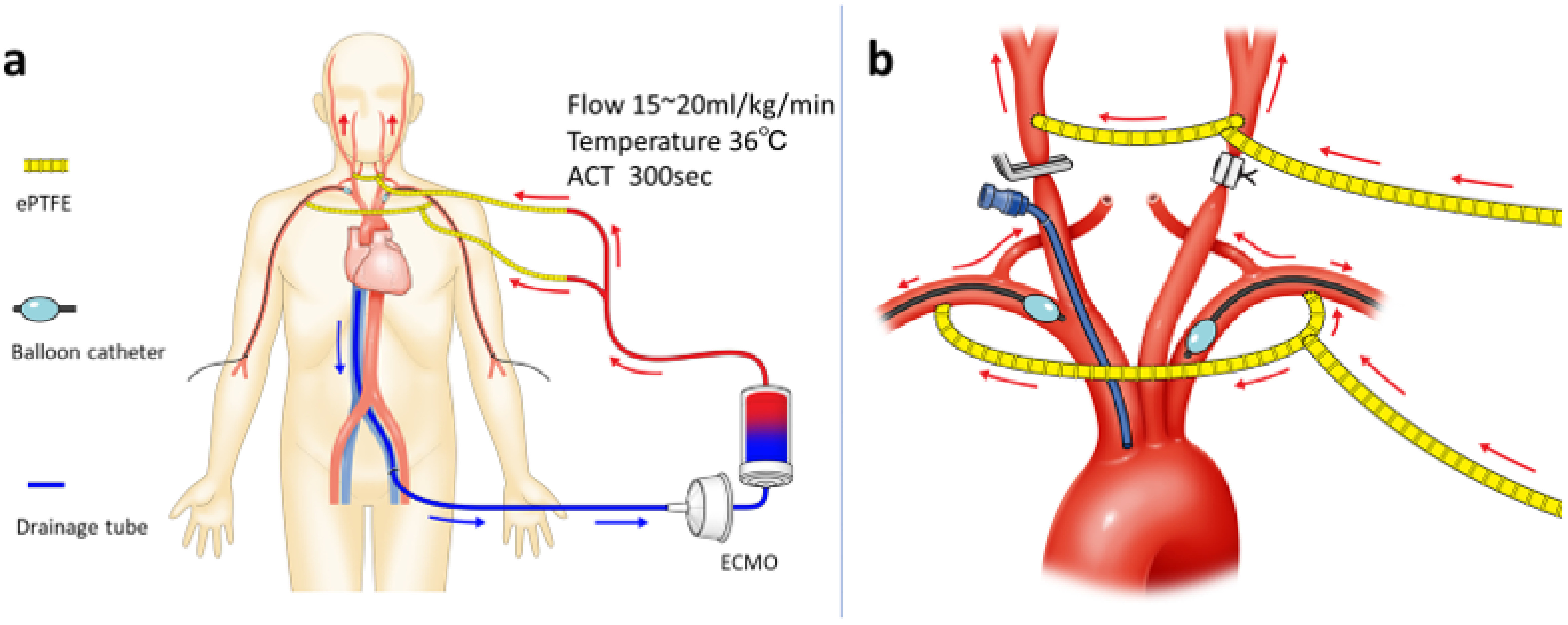
Fig. 1 Schematic of novel brain protection method. (**a**) Schematic of extracorporeal selective cerebral perfusion. (**b**) Precise blockage of arch branches. Bilateral subclavian arteries are blocked using balloon catheter, the left carotid artery is ligated with 3-0 Prolene, and the right carotid artery is clamped using DeBakey clamp forceps.

**Figure 2:**
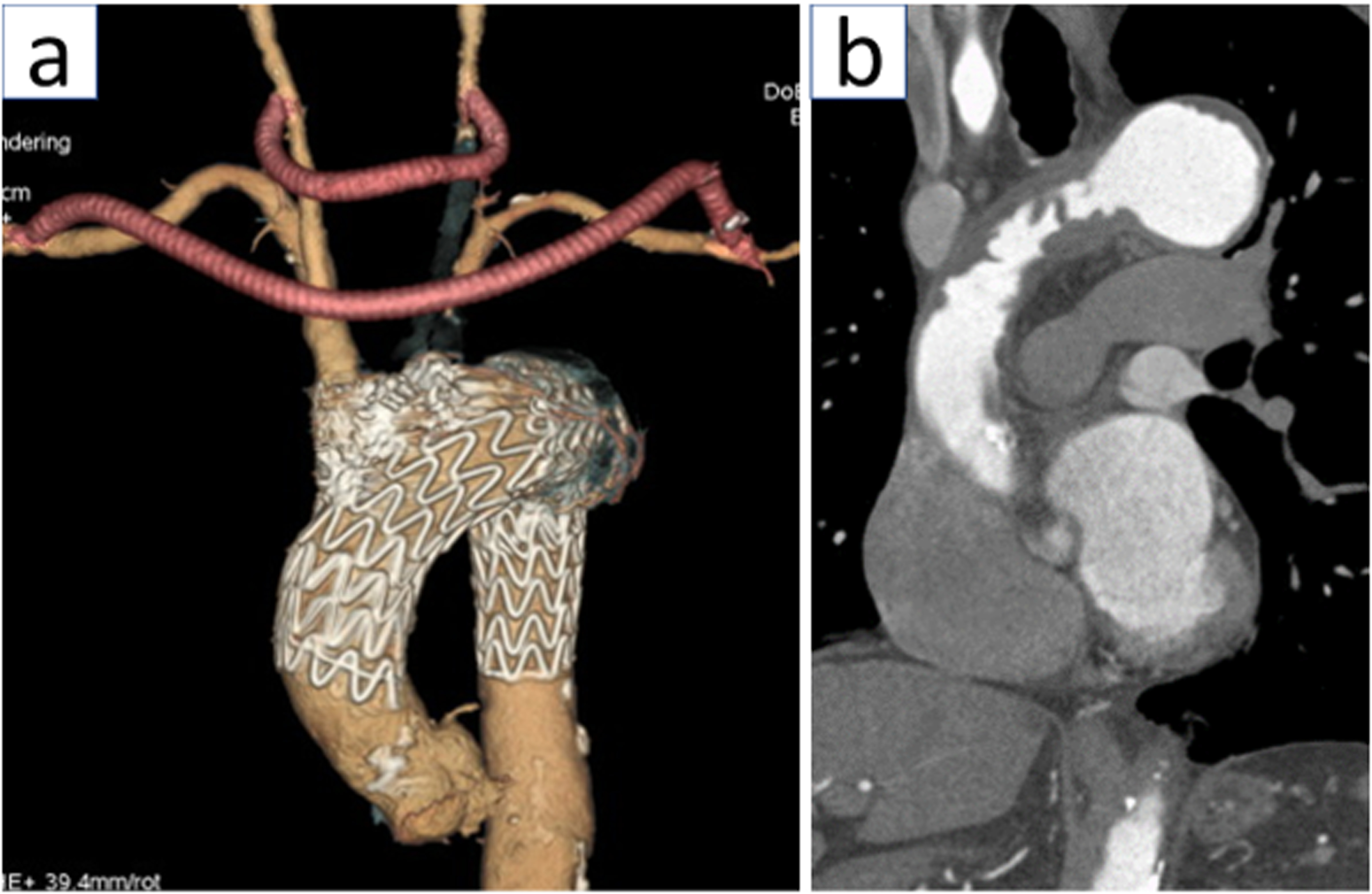
Fig. 2 Image of a practical case that underwent chimney thoracic endovascular aortic repair (TEVAR). (**a**) Three-dimensional image of completed chimney TEVAR with carotid–carotid bypass and axillo-axillary bypass. (**b**) Preoperative computed tomography sagital view. Grade IV atheroma was observed in the ascending aorta and aortic arch.

**Figure 1** presents the scheme of selective cerebral perfusion. **Figure 2a** presents an image of chimney TEVAR. The surgical techniques are detailed as follows.

### Establishment of carotid–carotid bypass and axillo-axillary bypass using two T-shaped expanded polytetrafluoroethylene (ePTFE) grafts

Two T-shaped ePTFE grafts were created by anastomosing ePTFE (ADVANTA VXT, Getinge Japan, Tokyo, Japan) with a diameter of 8 mm to ePTFE with a diameter of 7 mm in an end-to-side manner using the Gore-Tex Suture (CV-5, Japan Gore, Tokyo, Japan). The bilateral axillary arteries were exposed with a subclavian incision, whereas the bilateral carotid arteries were exposed with an incision made internal to the sternocleidomastoid muscle. After tunneling two T-shaped grafts under the skin, heparin (5000 units) was intravenously injected. The horizontal end, 7 mm in diameter, of the T-shaped graft was anastomosed with bilateral axillary arteries in an end-to-side fashion using polypropylene suture (6-0 Prolene, Ethicon Japan, Tokyo, Japan). Carotid–carotid bypass was established similarly. Graft-carotid artery anastomoses were established separately under 100% O_2_ ventilation.

After establishing the carotid–carotid bypass, the left carotid artery was closed using polypropylene suture (3-0 Prolene, Ethicon Japan, Tokyo, Japan) proximal to the graft anastomosed site. The vertical end of each T-shaped graft, 8 mm in diameter, was connected to the arterial line of ECMO (**Fig. 1**).

### Exposure of access route for TEVAR

Depending on the size of the stent graft required, the unilateral iliac or femoral artery was exposed. When the diameter or calcification of the femoral or external iliac artery was insufficient for the access route, the unilateral common iliac artery was exposed via the retroperitoneal approach, and a conduit was established by anastomosing a 10-mm knitted Dacron graft (Hemashield, Invervascular SAS, Paris, France) to the common iliac artery using 5-0 Prolene in an end-to-side fashion.

### Protection of the visceral and lower extremities

For cases in which the ostium could be selected endovascularly, the superior mesenteric artery and bilateral renal arteries were blocked using balloon catheters (Selecon MP Catheter II, TERUMO, Tokyo, Japan) that were inserted from the femoral artery contralateral side of the stent graft access route. Moreover, to protect the lower extremities from embolism, the superficial femoral arteries were blocked using DeBakey clamp forceps.

### Establishment of selective cervical extracorporeal circuit

A drainage tube was inserted from the left femoral vein and connected to the venous line of ECMO. The arterial line was connected to the vertical ends of the T-shaped grafts. Active coagulation time was maintained at >300 s. A 10-mm occlusion balloon (Python, Applied Medical, Santa Margarita, CA, USA) was advanced to the origin of bilateral subclavian arteries via the brachial arteries. The blood flow, transmitted through the arterial line connected to the two T-shaped grafts, was controlled at 15–20 mL/kg/min at a blood temperature of 36°C. While the extracorporeal circuit was running, the bilateral subclavian arteries and right carotid arteries were blocked; the subclavian arteries were blocked using balloon and the right carotid artery using clamp forceps. Since the left carotid artery was already occluded by suturing, cerebral perfusion and systemic perfusion were completely separated (**Fig. 1**). The regional cerebral oxygen saturation (rSO_2_) was monitored via near-infrared spectroscopy (NIRO-200NX, HAMAMATSU Photonics, Shizuoka, Japan).

### Pacing catheter

A pacing catheter (Bipolar pacing catheter, Nipro, Gifu, Japan) was inserted from the right femoral vein, and the tip was placed in the right ventricle. To avoid migration, rapid pacing (180 bpm, maximum of 20 s) was performed while deploying the stent graft in the ascending aorta and expanding the graft using a large balloon catheter (Trilobe balloon catheter II, Japan Gore, Tokyo, Japan) in the aorta.

### Deployment of the stent graft (Chimney technique)

**Figure 3:**
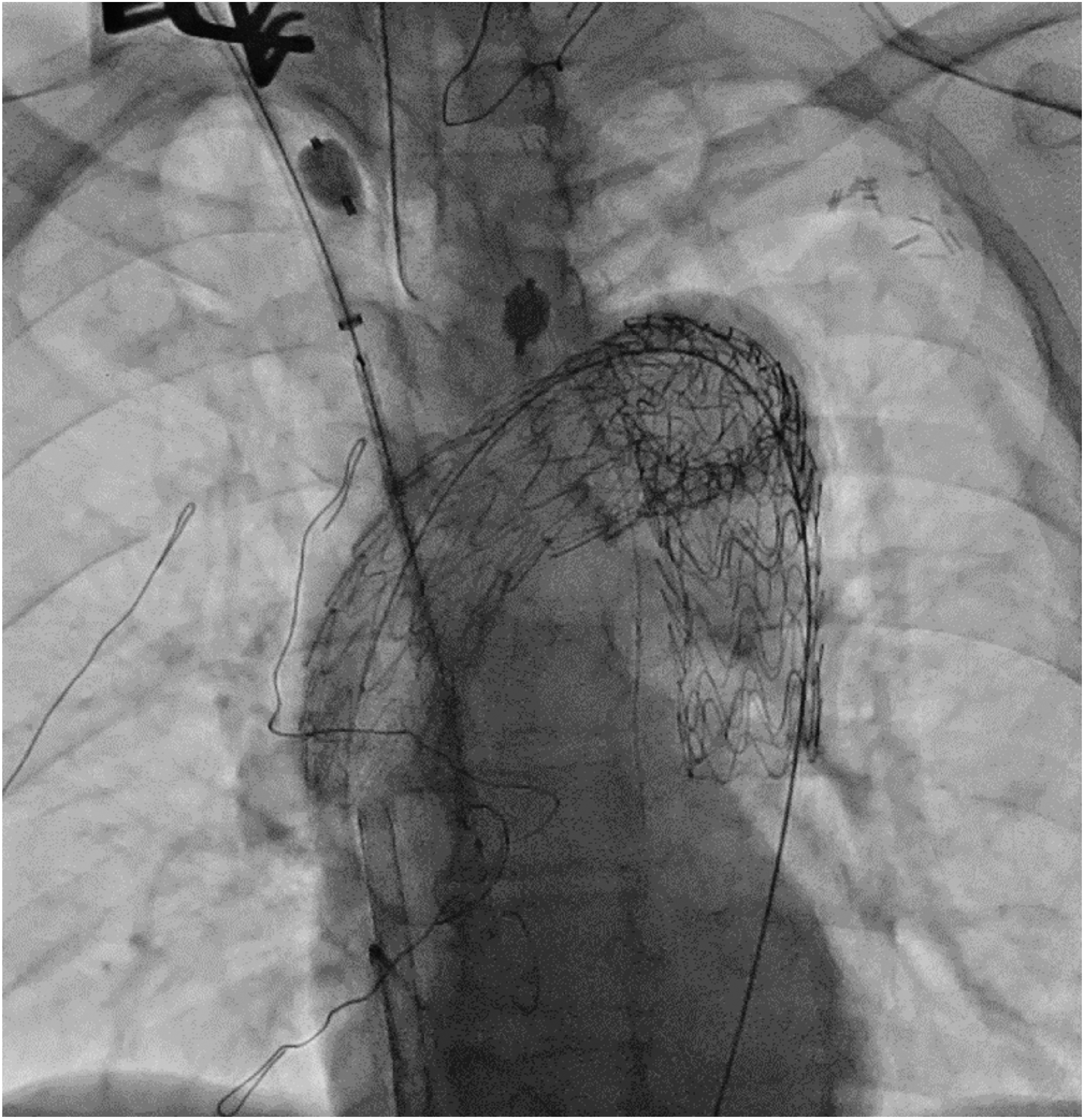
Fig. 3 Angiography during the deployment of the brachiocephalic chimney graft. During the deployment, the bilateral subclavian arteries and carotid arteries were perfused by ECMO, and cerebral circulation was totally independent from systemic circulation.

Initial angiography was performed using a pigtail catheter inserted from the access route. A 0.035-in guidewire (Radio Focus, Terumo, Tokyo, Japan) was used to guide the pigtail catheter. After confirming that cerebral perfusion was totally blocked from the systemic perfusion, a stiff guidewire was inserted into the aorta. The two main bodies of the stent graft (C-TAG, Japan Gore, Tokyo, Japan) were used in each patient. After deploying the first stent graft to the distal arch, the second stent graft was proceeded to Zone 0. Before deploying the proximal stent graft, the chimney graft (Excluder contralateral leg, Japan Gore, Tokyo, Japan) was inserted via the proximal right carotid artery. The proximal marker was confirmed in the ascending aorta, and the distal marker was confirmed in the brachiocephalic artery. The chimney graft and main body were deployed to match the proximal marker (**Fig. 3**). Rapid pacing was performed during the deployment. Subsequently, balloon expansion of the stent graft was performed. Expansion of the proximal portion was performed using the kissing technique under rapid pacing. A large balloon catheter (Trilobe balloon catheter II, Japan Gore, Tokyo, Japan) was used for the expansion of the main body, and a small balloon catheter (TMP SG balloon, Tokai Medical, Aichi, Japan) was used for the expansion of the chimney graft.

Besides the aforementioned parallel endograft chimney technique, the in situ fenestration technique was performed for selected patients according to the method reported by Hongo et al.^7)^

### Finishing extracorporeal circuit

Before declamping the clamp forceps in the right carotid artery, the sheath was evacuated from the artery, and the debris was flushed out from the hole. Subsequently, the forceps were removed, and the occlusion balloon in the right subclavian artery was deflated. The extracorporeal circuit was stopped, and cerebral circulation was reconnected with the systemic perfusion.

### Embolization of the gutter and left subclavian artery

Coil embolization of the gutter between the chimney and main body was performed. The approach was made via the proximal portion of the left carotid artery. Embolization of the bilateral gutters was confirmed. The left subclavian artery and left carotid arteries were embolized using coils and plugs. Angiography was performed to check for the remaining type II endoleaks.

### Finishing

Total exclusion of the aneurysm was confirmed via aortography along with the investigation of the endoleaks. The appropriate dose of protamine was administered. All the systems were evacuated, and the incisions were closed subsequently.

### Definitions and clinical follow-up

The primary outcomes of interest were death, cerebrovascular accidents, and major postoperative complications, such as bleeding that requires reoperation, acute renal failure that requires dialysis, infections, and pneumonia. Clinical follow-up data were routinely obtained via computed tomography (CT) at 7 days and 3–6 months after surgery to evaluate postoperative endoleaks.

## Results

### Preoperative atherosclerosis assessment via computed tomography

The severity of aortic atheroma was evaluated according to the modified CT grading scale for aortic atheroma, as described by Gutsche et al.^6)^: Grade 1, smooth and continuous aortic intimal surface; Grade 2, intimal thickening of 3–5 mm; Grade 3, atheroma protruding <5 mm into the aortic lumen; and Grade 4, atheroma protruding >5 mm into the aortic lumen and ulcerated or pedunculated (**Fig. 2b**). Grade 4 atheroma was observed in three patients (**Table 1**).

### Operative outcomes

Operative outcomes are presented in **Table 2**. Brachiocephalic artery graft was established using the described chimney technique in five patients and in situ fenestration technique in two patients. The overall operation time (mean±standard deviation) was 426±63 min. The overall selective extracorporeal brain perfusion time was 94±65 min; it was 59±16 min with the chimney technique and 216±48 min with the in situ fenestration technique. The carotid artery clamp time for establishing the bypass was 9±3 min. Transfusion was required in four patients.

**Table 2:** Table 2 Operative outcomes (n=7)

Variable	
Operation time (min)	426±63
SCP time (min)	
Overall	94±65
Chimney technique	59±16
In situ fenestration	216±48
Rt CA clamping time (min)	9±3
Lt CA clamping time (min)	9±2
Brachiocephalic artery graft (%)	
Chimney	5 (71)
In situ fenestration	2 (28)
Proximal Landing Zone (%)	
Zone 0	7 (100)
Transfusion required (%)	4 (57)
Death	0 (0)
CVA	0 (0)
Hoarseness	1 (14)
New-onset dialysis	0 (0)
Infection	0 (0)
Pneumonia	0 (0)
Prolonged ventilation (>48 h)	0 (0)
Reoperation for bleeding	0 (0)
Extubation (days)	1±0
Postoperative ICU stays (days)	2.7±0.8
Postoperative hospital stays (days)	15.8±1.8
Endoleak (%)	
Early endoleak (7 days after surgery)	2 (28)
Midterm endoleak (>3 months after surgery)	0 (0)
Aneurysm dilatation	0 (0)

Data expressed as n (%) or mean±standard deviation. SCP: selective cerebral perfusion; Rt: Right; Lt: Left; CA: carotid artery; CVA: cerebrovascular accident; ICU: intensive care unit

Postoperatively, all the patients were weaned from the respirator 1 day after surgery. The mean ICU stay was 2.7±0.8 days, and the hospital stay was 15.8±1.8 days. The mortality rate was 0%. No neurological complications were observed in any patient, except hoarseness due to left vocal cord paralysis in one patient.

On postoperative CT, trivial type I endoleak was observed in two patients. However, the endoleak was not observed during the follow-up. Moreover, there were no enlargements of aneurysms during the follow-up.

## Discussion

In the present study, no symptom of cerebral infarction was observed in any of the patients who underwent Zone 0 TEVAR with the novel selective cerebral perfusion with ECMO. This finding indicates that cerebral embolization due to scattering of the aorta was blocked using the novel method; therefore, the patients remained asymptomatic after surgery.

ECMO has the advantage of no concerns with regard to arterial plaques flowing into the cerebral circulation from the arterial line as the blood is originally supplied from the veins of the inferior vena cava. The idea of connecting the cardiopulmonary bypass to the branch of the cervical grafts was previously reported by Ryomoto et al. and Tsuda et al.; they reported a decrease in the incidence of cerebral infarction in Zone 1, 2, and 3 TEVAR.^4,9)^ Our method differs from theirs in that the right carotid and subclavian arteries were blocked, and the cerebral circulation was totally independent from the systemic circuit. Therefore, our method is applicable to the cases requiring Zone 0 proximal landing.

We blocked not only the carotid anterior circuit but also the vertebral posterior circuit from embolization. Ullery et al. reported that cerebral infarction involving the posterior circulation is related to higher morbidity and mortality than that of the anterior circulation.^9)^ We completely agree with this statement as embolic shower in the vertebral artery results in fatal ischemia in the brain stem and cerebellum. However, we assume that maintaining blood flow to the posterior circuit is not as important as blocking embolism. In case of a decrease in rSO_2_ in the anterior circulation during the extracorporeal support, we clamp the arterial line to the bilateral subclavian arteries and maintain the blood flow of the anterior circulation.

Precise attention has to be paid at the time of finishing the extracorporeal circuit. Debris may be trapped in the proximal portion of the block. Thus, it is important to avoid spattering of the plaques while declamping. In our method, no considerations are needed with regard to the plaques trapped in the left carotid and subclavian arteries as the left carotid artery is already occluded and the antegrade flow of the left subclavian artery is blocked by the main body of the stent graft. Therefore, the debris we should care about are those that flow into the brachiocephalic artery. First of all, we evaluate the occluding balloon at the site of just proximal to the right subclavian artery so that the plaque will not be trapped in the right subclavian artery and flow into the right carotid artery. The plaques trapped in the right carotid artery will be removed by flushing the blood out of the vessel from the hole where the sheath for the chimney graft was inserted. By declamping the clamp forceps after evacuating the sheath and flushing the blood, debris will not scatter to the brain.

A risk of embolization also underlies while establishing the carotid–carotid bypass. Careful examination of enhanced CT images is important to determine the adequate clamping site and safe procedure. When diffuse calcification is observed in the carotid artery and the risk of plaque exfoliation exists in either clamping site, we employ rapid pacing at the timing of clamping and avoid the plaque flowing into the brain.

No neurological complications were observed with extracorporeal circulation in either of the patients over a duration of 94±65 min. The flow and temperature of ECMO in our method are derived from our experience with total arch replacement.^1)^ The flow of 15–20 mL/kg/min from the selective cerebral perfusion system maintains the mean intracranial artery pressure at 60–70 mmHg. We maintained the temperature at 36°C because we believe that hypothermia is unnecessary to avoid brain damage under sufficient extracorporeal flow. During hypothermia, cerebral vascular resistance increases and perfusion worsens. It has been reported that the temperature imbalance during the rewarming phase induces brain damage.^1)^ In addition, contrary to open arch replacement, systemic perfusion is maintained with the natural circulatory system in our method. During TEVAR in our institute, systemic temperature usually drops down to 34.5°C–35.0°C naturally. If we perfuse the cerebrum with tepid blood flow, rewarming of the systemic temperature would be difficult, since the extracorporeal circuit consisted of small cardiopulmonary support.

There are some knacks in accomplishing chimney TEVAR with satisfying results. Chimney TEVAR has the negative potential of type Ia endoleaks.^2)^ In two of our patients, slight type Ia endoleak was observed on CT taken 7 days after surgery. However, the endoleaks resolved in the follow-up imaging, which is similar to the report of Zhao et al.^10)^ The important points for accomplishing the chimney technique are size selection, sufficient proximal landing zone, and elaborate filling of the gutter with coils. For size selection, the proximal main body should be the largest in the size applicable for the diameter of the native ascending aorta. The size of the chimney graft depends on the size of the brachiocephalic artery and does not have to be large. The distance of the proximal landing zone should be sufficiently long, at least more than 2 cm from the brachiocephalic artery. To deploy the main body as proximally as possible, we used C-TAG (Japan Gore, Tokyo, Japan). Since C-TAG’s proximal tip is the shortest of all available stent grafts, the risk of interference to the aortic valve is low. More importantly, embolization of the gutter is mandatory. If the approach to the bilateral gutters is not accomplished from the left carotid artery, we should not hesitate to approach from the left subclavian artery; this is why we embolize the left subclavian artery at the end of the procedure. Furthermore, sufficient proximal landing zone makes the gutter coiling safe since the risk of coils entering the aortic cavity decreases.

The limitation of this study was that postoperative cerebral magnetic resonance imaging was not performed. Tsuda et al. reported that asymptomatic solitary infarction may occur after TEVAR.^8)^ We cannot exclude the possibility of asymptomatic stroke. However, the absence of neurological deficit in seven consecutive patients indicates reliable clinical outcomes.

## Conclusion

This novel brain protection method with selective cerebral perfusion under percutaneous cardiopulmonary support blocks the brain from the scattering atheroma and helps avoid brain infarction in Zone 0 TEVAR.
